# Evaluating the intensity of fire at the Acheulian site of Gesher Benot Ya'aqov—Spatial and thermoluminescence analyses

**DOI:** 10.1371/journal.pone.0188091

**Published:** 2017-11-16

**Authors:** Nira Alperson-Afil, Daniel Richter, Naama Goren-Inbar

**Affiliations:** 1 Martin (Szusz) Department of Land of Israel Studies and Archaeology, Bar Ilan University, Ramat-Gan, Israel; 2 Department of Human Evolution, Max Planck Institute for Evolutionary Anthropology, Leipzig, Germany; 3 Freiberg Instruments GmbH, Freiberg, Germany; 4 Institute of Archaeology, Hebrew University of Jerusalem, Jerusalem, Israel; Max Planck Institute for the Science of Human History, GERMANY

## Abstract

This manuscript presents an attempt to evaluate the intensity of fire through spatial patterning and thermoluminescence methodology. Previous studies of Layer II-6 Level 2 at the Acheulian site of Gesher Benot Ya‘aqov suggested that hominins differentiated their activities across space, including multiple activities around a hearth reconstructed on the basis of the distribution of burned flint artifacts. A transect of ~4 m was extended from the center of the reconstructed hearth of Level 2 to its periphery in order to examine the intensity of fire. Burned and unburned flint microartifacts were sampled along this transect. The results of earlier and current thermoluminescence (TL) analysis demonstrate a general agreement with the macroscopic determination of burning, indicating that the possibility of misinterpretation based on macroscopic observations is negligible. The TL signal from flint microartifacts close to the hearth’s center shows unambiguous signs of strong heating, whereas with increasing distance from the hearth the TL signal can be interpreted as a result of decreasing temperatures and/or shorter durations of exposure to fire in addition to a decreasing number of flints showing fire damage. Our study shows that TL analysis can identify some variation in fire intensity, which allows a more precise classification of burned flint microartifacts with respect to their heating history.

## Introduction

The use of fire by early hominins is considered a significant technological and cultural revolution, thus a variety of methods and techniques are used in the attempt to determine past heating and study early fire. The chemical and microscopic analyses of sediments, considered reliable by many, are not always feasible and spatial analysis of burned residues is often the most accessible and efficient way to determine heating, particularly where a durable component such as lithics is concerned [1]. Research at the Acheulian site of Gesher Benot Ya‘aqov (GBY) has revealed the presence of latent hearths, identifiable only through spatial analysis of burned and unburned flint microartifacts (2–20 mm) [1, 2]. Subsequently, thermoluminescence (TL) analysis provided independent verification of the macroscopically identified burning [3] and suggested that the combination of spatial data and TL methods can be utilized to evaluate the intensity of fire at the site. At GBY, despite the excellent state of preservation of organic material (macro and micro botanical remains, including charcoal), spatial analysis of burned and unburned flint microartifacts was found to be the most reliable method for the study of early use of fire, enabling the identification of the earliest use and control of fire in Eurasia [1, 2].

While spatial analysis uses the macroscopic dichotomy of burned vs. unburned to delineate the location of the hearths, in this study TL methods are used not only to support the spatial data, but also to refine the scale of burning intensity and to provide information for the interpretation of fire behavior within the hearth and its periphery.

### Gesher Benot Ya‘aqov

Gesher Benot Ya‘aqov (GBY) is an open-air waterlogged Acheulian site located in a lake margin environment of paleo-Lake Hula. Acheulian artifacts are embedded throughout the entire stratigraphic sequence, illustrating continual hominin occupations along the lake margin over a total duration of some 100 ka during the Lower and Middle Pleistocene (MIS 20–18) [4, 5, 6].

The cultural record comprises a superimposition of 15 rich archaeological horizons located above the Bruhnes-Matuyama Chron Boundary, all representing a lithic tradition of the large flake phase of the Acheulian techno-complex, with its typical handaxes and cleavers. Paleontological assemblages include a great variety of faunal species [7, 8, 9] as well as an extremely rich paleobotanical assemblage [10, 11, 12, 13]. Diverse multidisciplinary studies have demonstrated that the individual archaeological horizons were minimally disturbed by postdepositional processes and that the original spatial configuration of all components has been preserved (e.g. [1, 8, 14, 15, 16, 17, 18]).

Our assumption that the archaeological horizons of GBY almost entirely preserved the original spatial organization is based on different lines of taphonomic evidence. The GBY site provides a rich cultural stratigraphy of individual, sequential Acheulian archaeological sites, at times separated by sterile layers devoid of cultural remains. Moreover, each of these sites is a thin stratigraphic unit whose thickness never exceeds the largest object found in it (a knapped artifact, a large mammal bone, etc.). The presence of these distinct single occupations provides a unique opportunity for diachronic examination of human spatial behavior and study of the impact of postdepositional processes. For these purposes numerous studies were carried out. Analysis of the faunal remains, particularly mollusks, demonstrated that embryos of *Viviparus apameae galileae* and *Bellamya* sp. were preserved within their shells, showing that from a taphonomic perspective this population underwent minimal postdepositional disturbance [14, 19]. In further examples from the faunal assemblages, in individual cases of conjoinable bones (elephant and *Dama*-size animal) the original spatial distribution was almost perfectly preserved ([8] table 7.9 and details therein, [15]). In each of these individual cases belonging to different layers/sites, the distance between the conjoined bones was in the order of centimeters [15]. The excellent preservation of mammal bones enabled the identification of various procedures of carcass handling (bone damage patterns such as cut marks, hack marks, etc.) [8, 20]. Another indication of minimal taphonomic impact is the preservation of extremely fragile bird bones, particularly that of an undamaged swan (*Cygnus*) skull ([8]: table 7.9 and details therein). The paleobotanical assemblages, including macrobotanical remains (wood, bark, bushes, climbers, fruits, seeds, vegetables, and underground storage organs), provide an unprecedented case of excellent organic preservation testifying to rapid sedimentation of each of the individual sites [21]. In addition, the virtually undisturbed nature of the sediments and their associated cultural remains is reflected by the abundant presence of microartifacts, which rules out winnowing. Finally, microartifacts are often found clustered, indicating the location of different knapping areas (in the case of microartifacts of different raw materials) or the location of hearths (in the case of burned microartifacts). These spatial patterns all attest to the minimal taphonomic disturbance of the individual sites, which allows high-resolution spatial analysis.

### Layer II-6 Level 2

**Fig 1:**
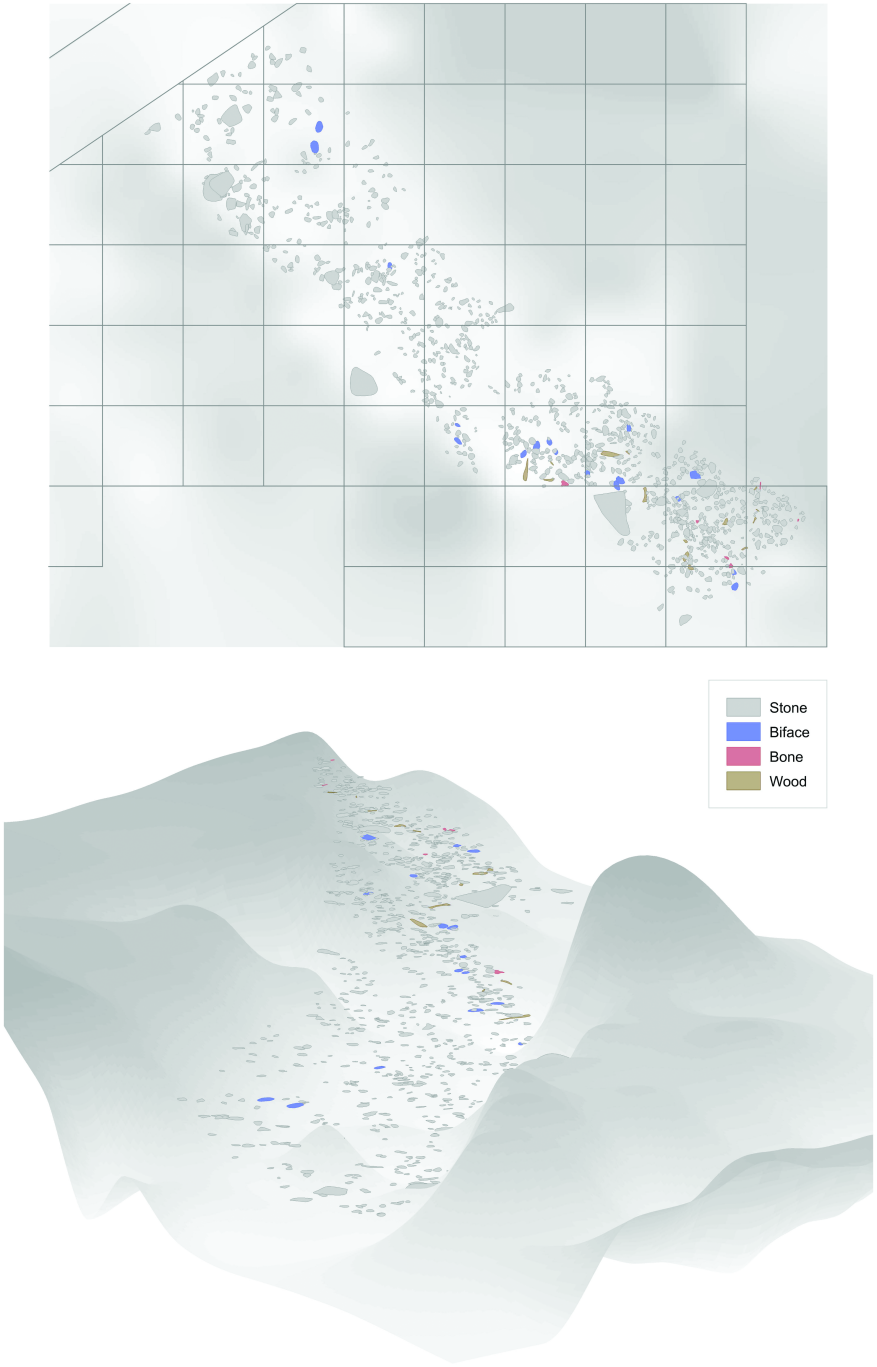
Field map of Layer II-6 Level 2: In plan view (top; grid is 1x1 m) and in its original tilted position (bottom; view to southeast); grey surface represents the topography of the excavated area, from light grey (low) to dark grey (high).

The archaeological horizon of Layer II-6 Level 2 has been shown to preserve clear spatial patterning of human activities, some in association with a hearth (Fig 1) [16]. Hence, this layer is used here for the spatial examination of the intensity of fire in the hearth and its vicinity. Layer II-6 Level 2 is one of the eight superimposed occupational levels recorded within Layer II-6.

The original spatial location of the archaeological finds was preserved within the sedimentary sequence of Layer II-6 due to the rapid sedimentation typical of a fluctuating low-energy lake margin environment. This is evidenced by the fresh preservation state of the archaeological material and the lack of winnowing. The thickness of the distinctly separated archaeological levels of Layer II-6 ranges between 0.06 and 0.18 m [17]. Layer II-6 Level 2, which is 0.12 m thick, was excavated over an area of 25.6 m^2^ and yielded an extensive botanical assemblage of wood, bark, fruits, seeds, and nuts, as well as highly diverse lacustrine and terrestrial faunal remains. A large lithic assemblage consisting of 79,670 microartifacts and 1,412 macroartifacts was recovered (Table 1).

**Table 1:** The lithic assemblage of Layer II-6 Level 2.

Category	Flint				Basalt	Limestone	Total
	unburned		burned				
	N	%	N	%	N	N	N
Microartifacts*	73,064	99.23	563	0.76	3,889	2,154	79,670
**FFT**^#^ artifacts*	300	99.00	3	0.99	771	15	1,089
**CCT**^##^ artifacts*	165	98.80	2	1.19	116	8	291
**Handaxes**	4	100.00	-		18	-	22
**Cleavers**	-		-		10	-	10
Pebbles*	792	99.74	2	0.25	875	107	1,776
**Total**	74,325	99.23	570	0.76	5,679	2,284	82,858

* The percentage of burned and unburned flint items is calculated within each lithic category;

^#^ Flakes and flake tools;

^##^ Cores and core tools

**Fig 2:**
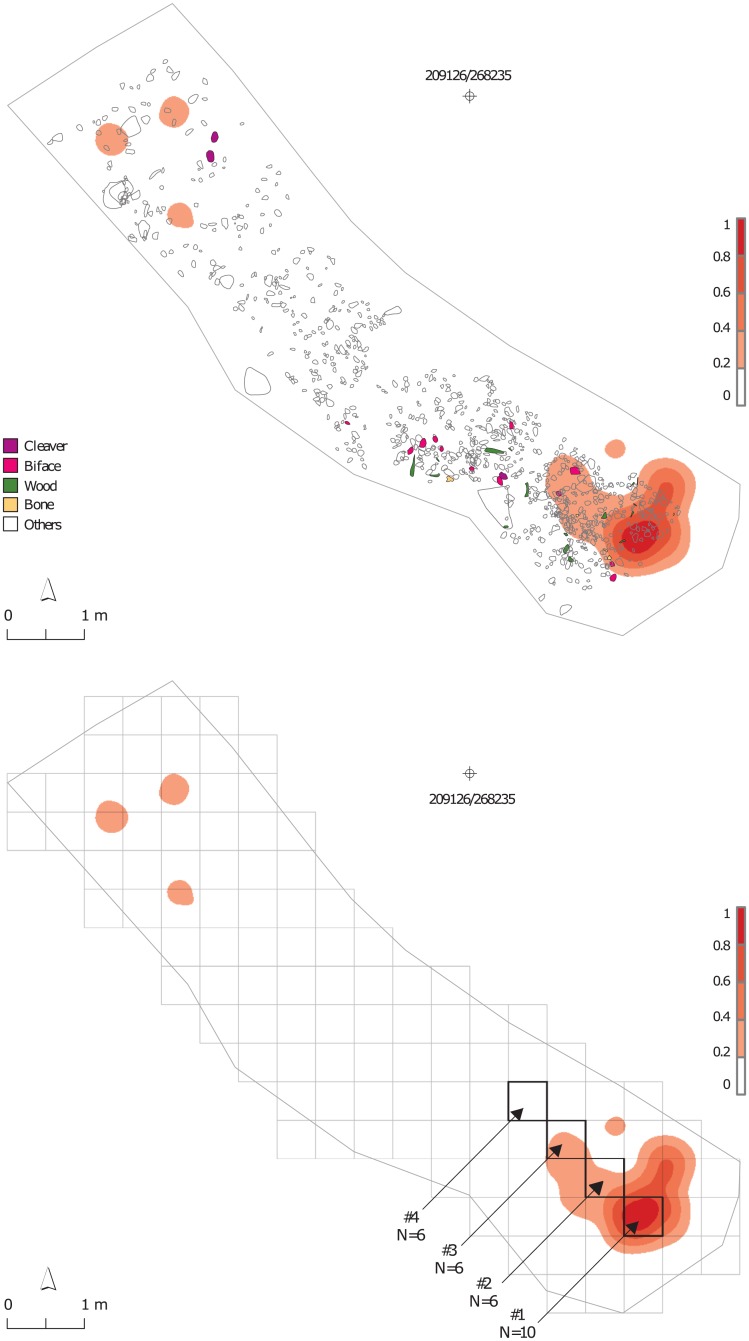
Field map of Layer II-6 Level 2 superimposed on the kernel density map (red scale) of burned flint microartifacts (N = 563) (top) and the location of the sampled sub-squares (#1–#4) along the transect (bottom; N indicates the number of flint microartifacts analyzed by TL).

Spatial analysis of this assemblage established strong evidence for the presence of activity areas, one being in the vicinity of a hearth [16]. The flints from this layer exhibit an overall very low frequency of burning, with only 0.76% of the microartifacts and 1.05% of the macroartifacts being burned. In addition, 0.25% of the flint pebbles show signs of burning (Table 1). Close to 60% of the burned flint microartifacts are clustered in an area of 3.25 m^2^ in the southeastern corner of the excavated surface (Fig 2).

Thus, we see a single high-density concentration of burned microartifacts in the southeastern corner of the excavated surface and a single high-density concentration of unburned flint microartifacts in the northwestern corner. A chi-square test confirms the significance of the apparent clustering of the burned microartifacts (Σχ^2^ = 913.27; *df* = 68; *p*<0.001; [1]). Furthermore, within the concentration of burned flint microartifacts, their percentage in the sub-square that encircles the highest-density kernel is 13% higher than what we would expect if the distribution of the burning were uniform. This pattern, together with the high significance of the standardized residual test of this sub-square (SR = 15.79; N [expected] = 20.5), points to this concentration being a major contributor to the observed clustering of burning ([1]: figs. 3.29–3.33).

Further spatial analysis of Layer II-6 Level 2 [16, 18] revealed differential use of space, whereby flint knapping was carried out mostly in the northwestern area and, to some extent, near the hearth, while the knapping of basalt and limestone was restricted to the hearth area. The hearth area was found to be spatially associated with segments of burned wood, particular tool types (e.g. percussive tools, chopping tools, side scrapers, end scrapers, and awls), and evidence of biface modification. In addition, the hearth served as a focal point for the processing and consumption of fish (Cyprinidae, carps) [16].

## Materials and methods

In order to evaluate the intensity of burning in the hearth and its periphery, our methodology combines spatial analysis and TL analysis.

### Spatial analysis

The methodology of spatial analysis of flint microartifacts was described in previous attempts to identify the presence and characteristics of hearths [1, 2, 22, 23, 24, 25]. The current study attempts to evaluate burning intensity and to provide information on fire behavior within the hearth and its periphery. Therefore, flint microartifacts from Layer II-6 Level 2 were sampled from a series of sub-squares forming a line (transect) from the center of the latent hearth to its periphery (Fig 2). As the latent hearth is in the southernmost corner of the excavated surface, the transect is oriented to the northwest. In addition, to allow systematic sampling, the transect follows the excavation grid obliquely through four sub-squares (Fig 2). Although numerous flint microartifacts were retrieved from each of these sub-squares (Table 2), the labor-intensive procedures for preparation of such small specimens for TL analysis, as well as the required measurement time, limited the number of flint that could be analyzed. Thus, 6–10 flint microartifacts were randomly sampled from each of the transect sub-squares, 10 from the sub-square in the center of the latent hearth, and 6 from each of its peripheral sub-squares (Fig 2). The sampling included equal numbers of burned and unburned specimens (Table 2), differentiated by macroscopic (visual) observation.

**Table 2:** Counts of flint microartifacts in Layer II-6 Level 2 and in the selected transect sub-squares, and the percentage of burning within them.

	N unburned		N burned		% burned
Layer II-6 Level 2	73,064		563		0.70
Transect	N	N sampled	N	N sampled	
**#1**	2,603	5	92	5	3.41
**#2**	866	3	28	3	3.13
**#3**	1,447	3	28	3	1.89
**#4**	835	3	5	3	0.59
**Total**	5,751	14	153	14	

### Thermoluminescence analysis

In previous studies, approximately 2,000 individual TL measurements were performed on various aspects of the rock material from GBY. These included Neutron Activation and HpGe γ-ray spectrometry of flint and basalt from the site, and UV-blue and orange-red signals of geological flint. The methodology of the previous and current analyses, including a detailed description of the development of the TL protocol, the parameters of TL procedures, and the bleaching experiments, has been published elsewhere [3], therefore only a short account is given here. Samples were, whenever possible, carefully stripped of the outer 1–2 mm of the entire surface with a water-cooled slow-speed saw. Careful crushing in a steel mortar with a hydraulic press was alternated with frequent sieving, followed by the removal of carbonates with 10% HCl. The coarse grain fraction (90–160 mm) was used for TL analysis.

The TL was measured for natural aliquots (NTL) and aliquots having received an additional dose from a beta source (NTL+beta) in the laboratory. The ratio of these signals over temperature were plotted for a heating plateau test [3, 26, 27, 28]. The presence/absence and maximum temperature of a constant ratio indicates the temperature and degree to which a sample has been heated in prehistory. In combination with the TL curve shape, an interpretation on the degree of heating can be provided [3, 26, 29, 30]. Based on previous results for GBY [3] and data from TL dating (e.g. [26, 27, 31]), the shape of the TL glow curve appears to be characteristic of well heated flint and the shape of TL curves can be used to estimate the degree of heating [3]. However, flint heated at low temperatures and/or for short durations is not easily detected by simple TL analysis and cannot be distinguished with confidence from samples that have been bleached during excavation. The latter is of special concern for translucent materials and/or thin samples. Hence, confident qualitative results can be provided only as heated (approximately >300°C), well heated (approximately >400°C), or not heated above approximately 250–300°C, provided that the sample has not been bleached; in the latter case the sample is additionally described as not heated but probably bleached. The category of not heated but bleached, or slightly heated is more frequent within the category of unburned flint microartifacts. In a previous analysis in which 83 macroscopically burned flint microartifacts underwent TL analysis, four were categorized as bleached and only a single artifact was assigned to the ambiguous category of not heated but bleached, or slightly heated ([3]: Table 2). Our previous studies [3, 24] demonstrated that when the TL signal is strong, bleaching can be considered negligible. A feeble TL signal, however, may be the result of exposure to atmospheric conditions during and/or after excavation (bleaching) or the result of slight heating, thus explaining the frequent occurrence of potential bleaching within the category of unburned flint microartifacts.

**Fig 3:**
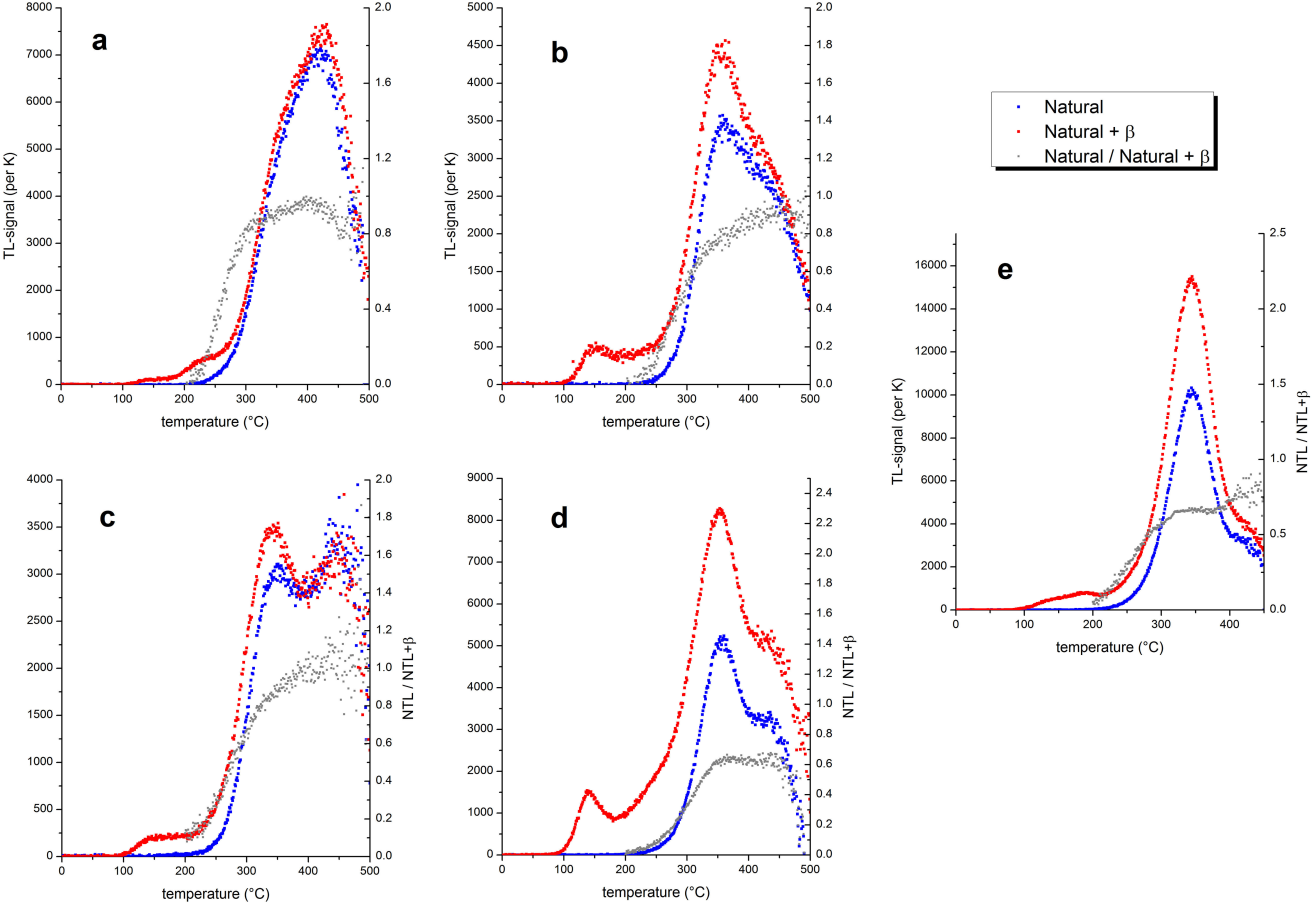
Examples of TL-curves and interpretation of curve shape, peak temperature and heating plateau test (a–e) after an artificial irradiation of 400 Gy (natural + β) on top of the natural dose (natural = NTL), ratio of NTL/NTL+β (heating plateau test). a) not heated; b) not heated but bleached, or slightly heated; c) slightly heated; d) heated; e) well heated.

Samples assigned to the category of slightly heated show a differentiation of TL peaks that may be caused by heating, but a signal increase by artificial irradiation because of recent bleaching cannot entirely be ruled out. Fig 3 shows examples of TL curves of natural flint (red), after additional artificial irradiation (blue), and heating plateau test results (grey) together with the interpretation of the presence/absence and interpretation of the degree of heating/bleaching. Samples which had not been heated (Fig 3a) lack an increase of the natural TL signal after artificial irradiation, do not provide a heating plateau (constant ratio) and only show a geological TL-signal with high temperature TL-peaks. Low temperature heating and/or bleaching results in a slight increase of the TL-signal after artificial irradiation (Fig 3b), but lack a heating plateau and show the presence of the geological TL-signal at high temperatures (≥ 400°C). The geological TL is still pronounced after slight heating to low temperatures (Fig 3c), but a TL-peak, which can be increased by artificial irradiation, is visible. Heating to temperatures well above 300°C allows a pronounced increase of the TL-signal after artificial irradiation, with a TL-peak in the temperature region of 350°C (at 5K s^-1^ heating rate) and a good heating plateau, but with the geological TL still well visible (Fig 3d). Well heated samples have experienced temperatures ~400°C or more, which results in a well-defined heating plateau, a pronounced TL-peak at ~360°C and little (negligible) geological TL-signal at very high temperatures (Fig 3e).

The analyzed flint microartifacts (with their catalogue numbers) are listed in Table 3. The microartifacts were destroyed due to the TL method of analyses. The GBY lithic assemblages are stored at the Institute of Archaeology, The Hebrew University of Jerusalem, and are accessible for scientific studies.

**Table 3:** The results of TL qualitative analysis of visually unburned (a) and visually burned (b) microartifacts. The approximate surface removal is given in mm, in order to provide an idea of the likelihood of bleached material being included in the analysis. See Fig 2 for transect location. TL qualitative also marked in color.

Transect section	No.	Surface removal	TL qualitative	
***a*. *visually unburned microartifacts***				
**#1**	559	~ 1		not heated but bleached
	560	< 1		not heated but bleached, or slightly heated
	561	0.0		not heated but bleached
	562	~ 1		slightly heated
	563	~ 1		not heated but bleached, or slightly heated
***#2***	567	~ 1		not heated but bleached, or slightly heated
	568	0.0		not heated
	569	< 1		not heated
***#3***	572	0.0		not heated but bleached, or slightly heated
	573	0.0		not heated but bleached
	574	0.0		not heated but bleached, or slightly heated
***#4***	106	2.0		not heated but bleached, or slightly heated
	578	~ 1		not heated
	579	1.5		not heated but bleached
***b*. *visually burned microartifacts***				
**#1**	557	~ 1		well heated
	558	1.5		heated
	104	2.0		well heated
	555	0.0		well heated
	556	0.0		well heated
**#2**	564	0.0		well heated
	565	0.0		heated
	566	0.0		not heated but bleached
***#3***	571	0.0		well heated
	105	2.0		heated
	570	< 1		heated
***#4***	575	~ 1		not heated but bleached, or slightly heated
	576	0.0		heated
	577	~ 1		slightly heated

## Results

The samples of burned and unburned flint microartifacts from Layer II-6 Level 2 that were tested for heating, and the results of their TL qualitative, are presented in Table 3. Based on the approach described above, six categories were identified, reflecting not only the intensity of heating (not heated, slightly heated, heated, well heated) but also the extent of confidence (not heated but bleached, not heated but bleached, or slightly heated). The purpose here is not to provide unequivocal results but rather trends for interpretation.

**Fig 4:**
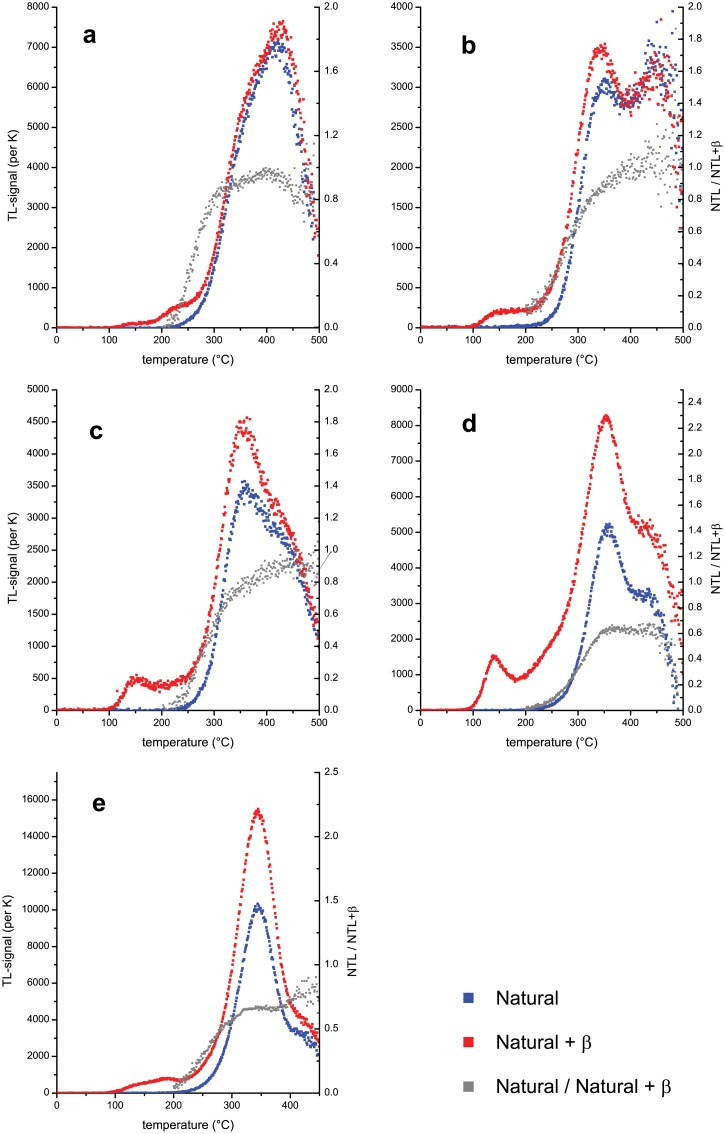
Schematic illustration of the results of TL qualitative analysis in comparison with the macroscopic (visual) classification of burned and unburned flint microartifacts; based on the TL results listed in Table 3, which in turn are based on the interpretation of TL heating plateaus and TL curve shapes as exemplified in Fig 3.

The results of the TL analysis are largely in agreement with the visual observations, and though the current sample is small, similar results were obtained previously [3]. The most important contribution of the current study is in achieving the spatial association between the hearth and the varying burning intensity of the flint microartifacts. While our macroscopic identification of burning cannot make a distinction between different intensities of exposure to fire, the TL analysis was able to demonstrate that fire intensity appears to decrease with distance from the hearth’s center. Within the area closest to the hearth’s center (transect #1), four of the five burned microartifacts were categorized as well heated, while within the area farthest away (transect #4), none of the three burned microartifacts was found to be well heated (Fig 4).

## Discussion

The results of this study show that the burning intensity of lithic microartifacts as determined by TL analysis is correlated with their distance from the point of highest concentration of heated material (the hearth). With distance from the hearth, flint microartifacts are generally less heated than in the hearth’s center, where they are most frequently found to be well heated. Such a resolution with respect to changing intensities of the burning of lithics in relation to combustion features has never been reported archaeologically. Similar spatial patterns are reported in the experimental study of Sérgant et al. [32], who attempted to measure the radiant heat of an open fire and to quantify its impact on surrounding artifacts. The experiment demonstrated that “… only those artefacts which were in direct contact with the fire were heated to a temperature above 300°C and showed heat damage. Artefacts lying outside the hearth, even those immediately bordering the hearth, were not affected by the heat at all. This is explained by the fact that immediately outside the hearth the temperature drops drastically” ([32]:1001). Our study provides results on archaeological material that support this experimental study, and further enlarges the scope of the data through the advantages provided by the application of the TL method showing a finer gradient of heating then that visible with the naked eye. This method allows an interpretation of different burning intensities that is macroscopically difficult to achieve, illustrating the spatial complexity of the hearth and its surroundings. Our results highlight two aspects:

1. With distance from the hearth, the general percentage of burning amongst microartifacts decreases (Table 2).
2. With distance from the hearth, the intensity of fire decreases, resulting in less intensively burned microartifacts (Table 3).

While the experiments of Sérgant et al. [32] demonstrated that microartifacts outside the hearth were not affected by the burning, our results show that burned microartifacts do occur outside the hearth, though in decreasing and significantly lower frequencies.

This difference may be the result of fracturing of the flint in response to the fire, as demonstrated experimentally: “small splinters (mainly pot-lids) of the artefacts were ejected from the hearth, as far as 2.5–3 m away” [32:1001].

Such patterns can be identified only in sites where the depositional record is essentially undisturbed by postdepositional taphonomic agents and has retained its original spatial configuration. GBY is an example of conditions in which individual, well-defined occupational horizons, preserving evidence of hearths, can be identified (see Materials and methods). Such depositional conditions are extremely rare in the Levant, and most of the evidence of fire originates in cave sequences. These include burned flint (e.g. Amud Cave [33]; Tabun Cave [34]), bone (Qesem Cave [35, 36]; Amud Cave [37]), and different sedimentological phenomena including hearths, cemented hearths, ashes, and siliceous aggregates (Hayonim and Kebara Caves [38, 39, 40, 41, 42]; Tabun Cave [43, 44]; Qesem Cave [45, 46]; Amud Cave [47]). The above survey represents some of the latest advances made in the study of fire use in Lower (Acheulian and Acheulo-Yabrudian) and Middle (Mousterian) Paleolithic cave sites (but see the Mousterian case of Nesher Ramla [48]). Cave sites differ drastically from open-air sites in that they are characterized by long stratigraphic and cultural sequences in which most of the deposits are anthropogenic. Various micromorphological studies have provided evidence for extensive anthropogenic fire-related activities at these cave sites. Whenever examined, the sequences of the end of the Lower Paleolithic and the entire Middle Paleolithic are characterized by a multitude of hearths and to a large extent by products of burning that form the cave deposits (e.g. Qesem, Kebara, Hayonim, Tabun Layers C and B, Amud, and Misliya Caves, to mention only the most extensively published ones). Clearly, in addition to the various types, forms, and sedimentological characteristics of hearths (color, texture, size, and cementation), other indications of fire, such as burned lithics, fauna, and flora, occur as well. However, although it is evident that fire was used intensively in the caves, it is in most cases impossible to distinguish individual occupational events from the general palimpsests of the cultural and sedimentological record. Thus, despite the wealth of evidence, these cave sequences lack the necessary resolution for interpreting the spatial organization of the hearths and associated activities within distinct occupational horizons (but see Abric Romaní [49]).

The cultural record of GBY is totally different from that revealed in cave sequences. The major difference stems from the identification of superimposed occupational events that are each a single artifact thick, preserving the spatial organization of the hearths and their associated cultural remains. These spatial associations include charred wood, crab pincers, concentrations of fish remains, flakes from the finishing stages of biface modification, percussive tools, and other particular tool categories [16]. The current study was able to provide even more detailed information on the distribution of fire-damaged artifacts from the perspectives not only of their spatial patterning but also of the degree of burning. Such hearth-related patterns are of a much higher resolution than those preserved in other Lower or Middle Paleolithic sites (excluding, e.g., the Mousterian site of Far‘a II [50, 51]). From the Upper Paleolithic onward one can find parallels (e.g. [32, 52, 53, 54, 55]) in which recorded clusters of burned microartifacts are interpreted as the remnants of hearths with spatial patterns that resemble in character and resolution those observed for GBY.

Further archaeological evidence is limited, since the prehistoric record is highly fragmented and long cave sequences lack the required resolution. Our study demonstrates that the combination of spatial and TL analyses of fire damaged artifacts can contribute much to sites lacking clear components indicative of hearths such as burned sediments, ashes, charcoal, burned bones, etc.

It seems clear that the hearth-related spatial patterns identified at GBY and at Upper Paleolithic and younger sites, as well as in experimental studies, were a common pattern, distributed over vast geographical zones and along an immense time trajectory, from which we see only a very partial picture. This is supported by the nearly universal character of similar hearth-related spatial patterns recorded for a variety of present-day hunter-gatherers (e.g. [9, 56, 57, 58, 59, 60]), which suggests that such behavior is not restricted to modern humans.
