# Correction: Evaluating the intensity of fire at the Acheulian site of Gesher Benot Ya'aqov—Spatial and thermoluminescence analyses

**DOI:** 10.1371/journal.pone.0190804

**Published:** 2018-01-02

**Authors:** Nira Alperson-Afil, Daniel Richter, Naama Goren-Inbar

Fig 4 is incorrect. Please view the correct Fig 4 here.

**Fig 4:**
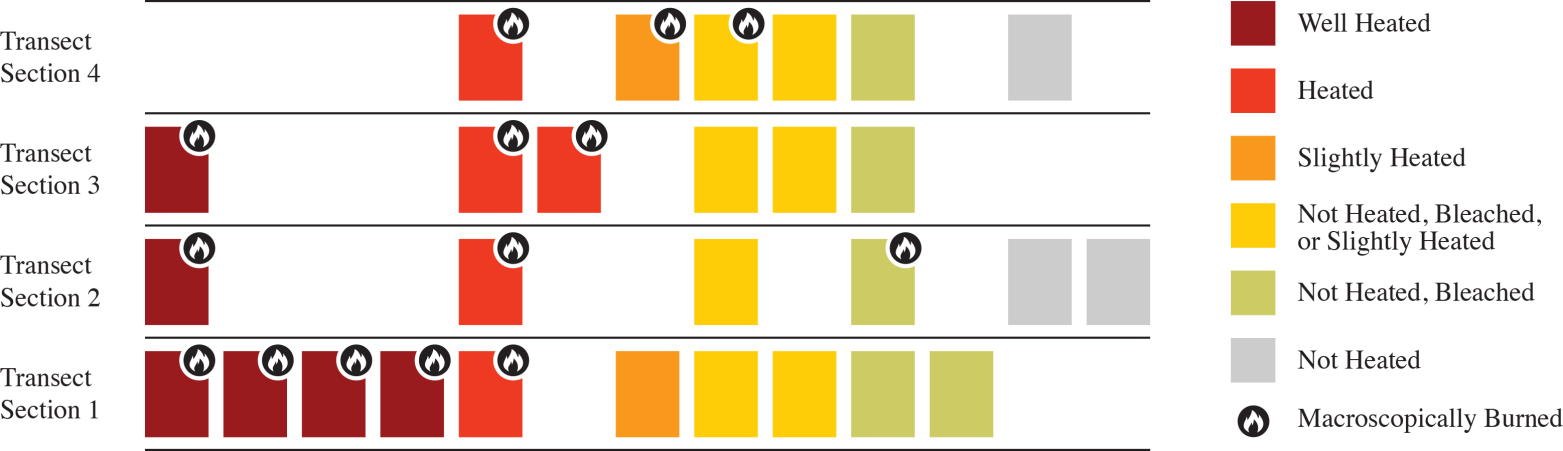
Schematic illustration of the results of TL qualitative analysis in comparison with the macroscopic (visual) classification of burned and unburned flint microartifacts; based on the TL results listed in Table 3, which in turn are based on the interpretation of TL heating plateaus and TL curve shapes as exemplified in Fig 3.
